# Subset selection of high-depth next generation sequencing reads for *de novo *genome assembly using MapReduce framework

**DOI:** 10.1186/1471-2164-16-S12-S9

**Published:** 2015-12-09

**Authors:** Chih-Hao Fang, Yu-Jung Chang, Wei-Chun Chung, Ping-Heng Hsieh, Chung-Yen Lin, Jan-Ming Ho

**Affiliations:** 1 Institute of Information Science, Academia Sinica, Taipei, Taiwan; 2 Department of Computer Science and Information Engineering, National Taiwan University, Taipei, Taiwan; 3 Research Center for Information Technology Innovation, Academia Sinica, Taipei, Taiwan

## Abstract

**Background:**

Recent progress in next-generation sequencing technology has afforded several improvements such as ultra-high throughput at low cost, very high read quality, and substantially increased sequencing depth. State-of-the-art high-throughput sequencers, such as the Illumina MiSeq system, can generate ~15 Gbp sequencing data per run, with >80% bases above Q30 and a sequencing depth of up to several 1000x for small genomes. Illumina HiSeq 2500 is capable of generating up to 1 Tbp per run, with >80% bases above Q30 and often >100x sequencing depth for large genomes. To speed up otherwise time-consuming genome assembly and/or to obtain a skeleton of the assembly quickly for scaffolding or progressive assembly, methods for noise removal and reduction of redundancy in the original data, with almost equal or better assembly results, are worth studying.

**Results:**

We developed two subset selection methods for single-end reads and a method for paired-end reads based on base quality scores and other read analytic tools using the MapReduce framework. We proposed two strategies to select reads: MinimalQ and ProductQ. MinimalQ selects reads with minimal base-quality above a threshold. ProductQ selects reads with probability of no incorrect base above a threshold. In the single-end experiments, we used *Escherichia coli *and *Bacillus cereus *datasets of MiSeq, Velvet assembler for genome assembly, and GAGE benchmark tools for result evaluation. In the paired-end experiments, we used the giant grouper (*Epinephelus lanceolatus*) dataset of HiSeq, ALLPATHS-LG genome assembler, and QUAST quality assessment tool for comparing genome assemblies of the original set and the subset. The results show that subset selection not only can speed up the genome assembly but also can produce substantially longer scaffolds. Availability: The software is freely available at https://github.com/moneycat/QReadSelector.

## Background

With the introduction of next-generation-sequencing technology, a vast amount of sequencing data can be generated in a short period of time. A major application in genome sequencing is *de novo *assembly, which aligns overlapping reads into super-sequences known as contigs and uses paired-end (PE) reads to further connect contigs into scaffolds [1]. To produce longer contigs and scaffolds, sequencing data with sufficient sequencing depth and low error rate are required. However, DNA sequencing reads from Illumina sequencers have previously generated errors at the rate of 0.5-2.5% [2], forcing researchers to develop various error correction algorithms in order to be able to use as many sequencing reads as possible. Recently, state-of-the-art high-throughput sequencers, such as the Illumina MiSeq series, have been reported to generate sequencing reads of around 2500x sequencing depth in small genomes, with >80% of bases above Q30 [3,4]. Another example is the Illumina HiSeq 2500 that is capable of generating up to 1 Tbp per run with >80% bases above Q30 [5]. Sequencing depth of the HiSeq data is often 100x or more for large genomes. The availability of such high sequencing depth and high-quality reads leads us to wonder if it is possible to select useful reads and read pairs from the original sequencing data, in order to assemble genomes without affecting assembly results or with even better results.

## Results

### Datasets and preprocessing

We downloaded two genome sequencing datasets, *Escherichia coli *MG1655 and *Bacillus cereus *ATCC 10987, from Illumina's MiSeq Scientific Data [4] (Table 1). Each dataset was sequenced on the MiSeq System, using the new MiSeq Reagent Kit v3 with a read length configuration of 2 × 300 bp. As shown in Table 1, both datasets have coverage of above 2500x. In addition, more than 80% of the bases are above Q30 for each dataset. The base quality score distribution and cumulative distribution of the two datasets are given in Additional files 1 and 2. The complete genomes from NCBI library are used for evaluation.

**Table 1:** The sequencing datasets used in the experiments.

Dataset	1	2	3
Species^11^	***E. coli***	*B. cereus*	Grouper

Genome size	4.6 Mbp	5.2 Mbp	~1.1 Gbp^2^

Read length	2 × 300 bp	2 × 300 bp	2 × 200 bp

Mean quality score	34	34	35

% Bases with quality score > 30	83%	85%	92%

Depth	2853x	2669x	~110-120x^2^

^1 ^The full scientific names of those species are *Escherichia coli*, *Bacillus cereus *and *Epinephelus lanceolatus*.

^2 ^Those are estimated values by ALLPATHS-LG, because the complete reference genome is not yet available.

To test subset selection for more complex genomes, we included the grouper NGS data, generated by HiSeq 2500, as the third dataset (Table 1). The grouper dataset consist of two PE libraries with a read length configuration of 2 × 200 bp and insert lengths 400 bp and 500 bp. The two libraries are similar in size and have the total size of 125G bp after adaptor and quality trimming by Trim Galore scripts [6]. The base quality score distribution and cumulative distribution of the grouper dataset are given in Additional file 3. In addition, there are five mate-pair libraries, with insert lengths ~2K, ~4K, ~6K, ~8K, ~10K bp, of the grouper. The size of each mate-pair library is ~4.4G bp. Note that the grouper dataset is sequenced by Prof. Lin's team (coauther of this paper) and is under preparation for publication.

The sequencing data were stored in FASTQ format, which provides information on the sequence identifier, read sequence and quality values for each base. The quality values are in ASCII format, and can be transformed into a probability *p*, which indicates the probability of the corresponding base call being incorrect. The quality value information contained in FASTQ files enables the selection of reads based on quality values. In order to process large-scale datasets more quickly, we developed preprocessing and analytic programs using Hadoop [7] and MapReduce [8] framework. The FASTQ format were converted into the *key*-*value *format before further processing, where the *key *field is the read identifier, and the read sequence and quality values are put as two fields of the *value*.

### Subset selection for single-end reads

Here, we propose two strategies to select a subset of reads based on quality value of each base. We use Velvet [9] to assemble the subset of single-end reads with k-mer size 221 (the reason is given in Additional file 4).

#### MinimalQ

**Figure 1:**
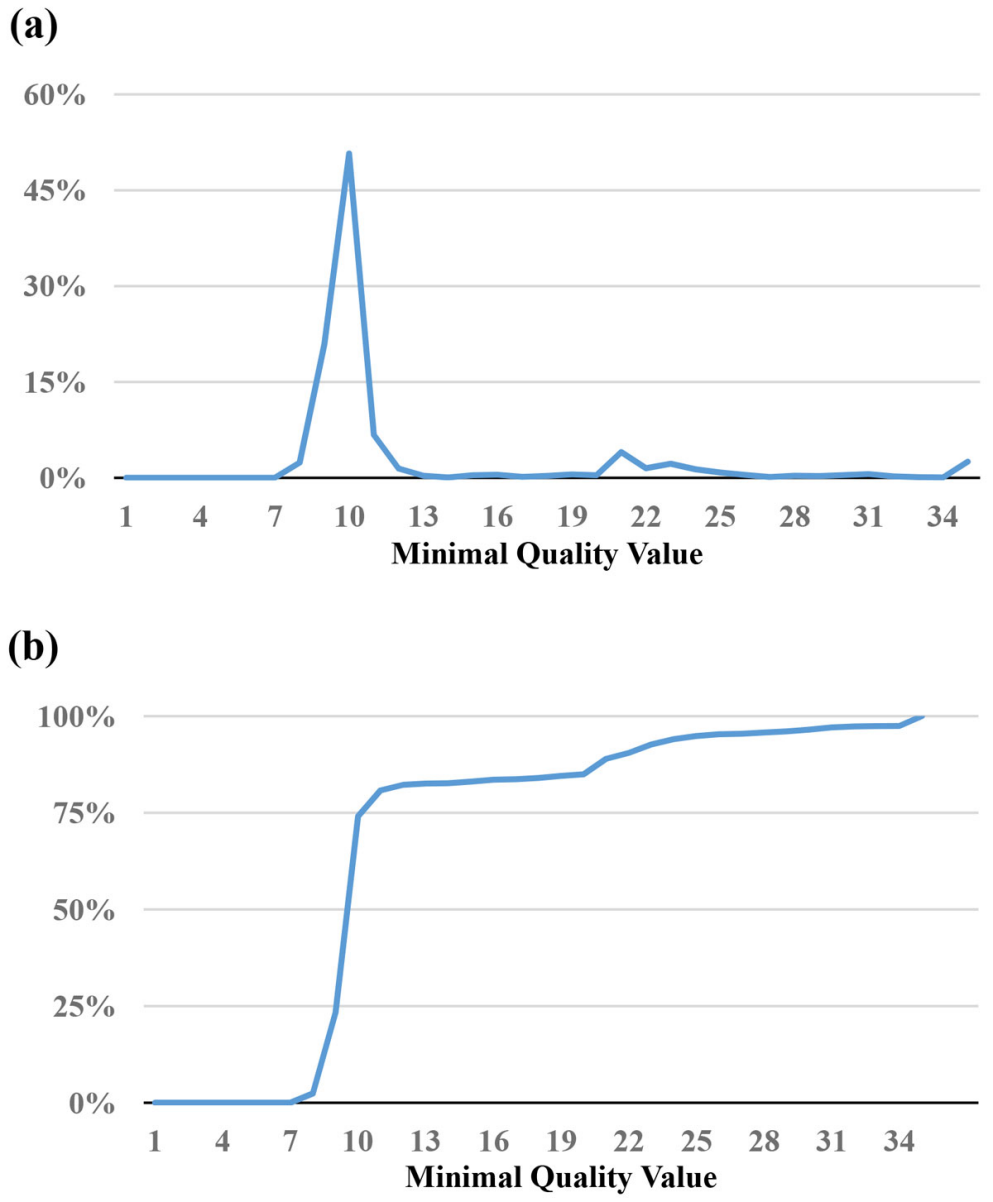
**Statistics of minimal quality value for the reads in the *E. coli *dataset**. (a) The percentage of reads for a minimal quality value. (b) The cumulative percentages of (a).

**Figure 2:**
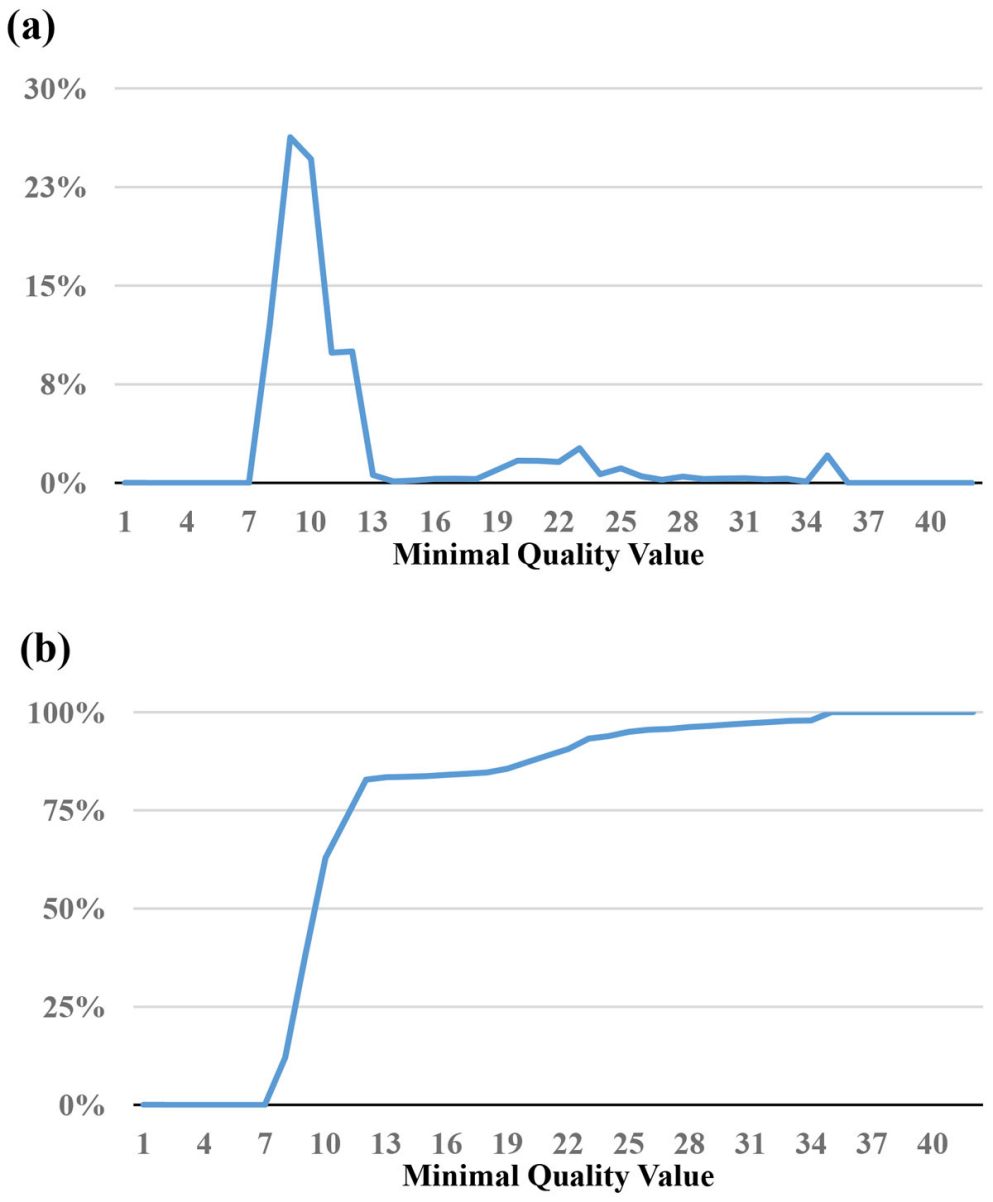
**Statistics of minimal quality value for the reads in the *B. cereus *dataset**. (a) The percentage of reads for a minimal quality value. (b) The cumulative percentages of (a).

Since the base with the lowest quality value is most likely to cause misassembled contigs, the *MinimalQ *strategy identifies the minimal quality value of each read, and sets a threshold of selecting reads with minimal quality value no smaller than the threshold. As shown in Figure 1a and Figure 2a, although both the *E. coli *and *B. cereus *datasets have bases quality values of 80% or above at Q30 (Table 1), the *E. coli *dataset has a peak at minimal quality value 9 with 51% (Figure 1a), and the *B. cereus *dataset at minimal quality value 8 with 26% (Figure 2a) in terms of reads' minimal quality statistics. This suggests that within these subsets of reads, although the other bases are correct, a few bases with low quality represent potential candidates that make reads unable to align. Thus, it is reasonable to filter out reads with low minimal quality value. The percentage of reads filtered out is shown in Figure 1b and 2b. For example, if we set 10 as the threshold, 81% of the *E. coli *reads and 73% of the *B. cereus *reads were filtered out.

#### ProductQ

**Figure 3:**
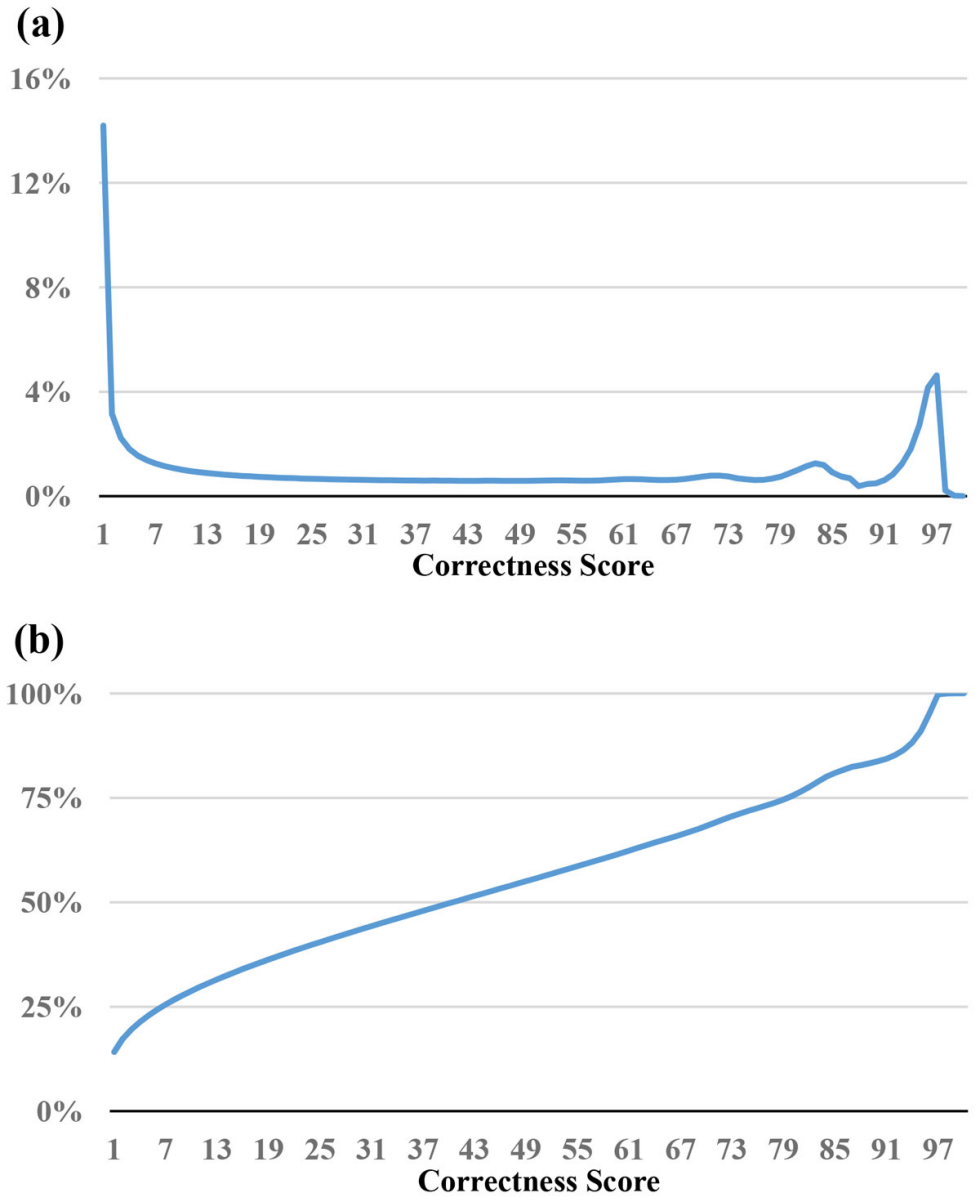
**Statistics of correctness score for the reads in the *E. coli *dataset**. (a) The percentage of reads for a correctness score. (b) The cumulative percentages of (a).

**Figure 4:**
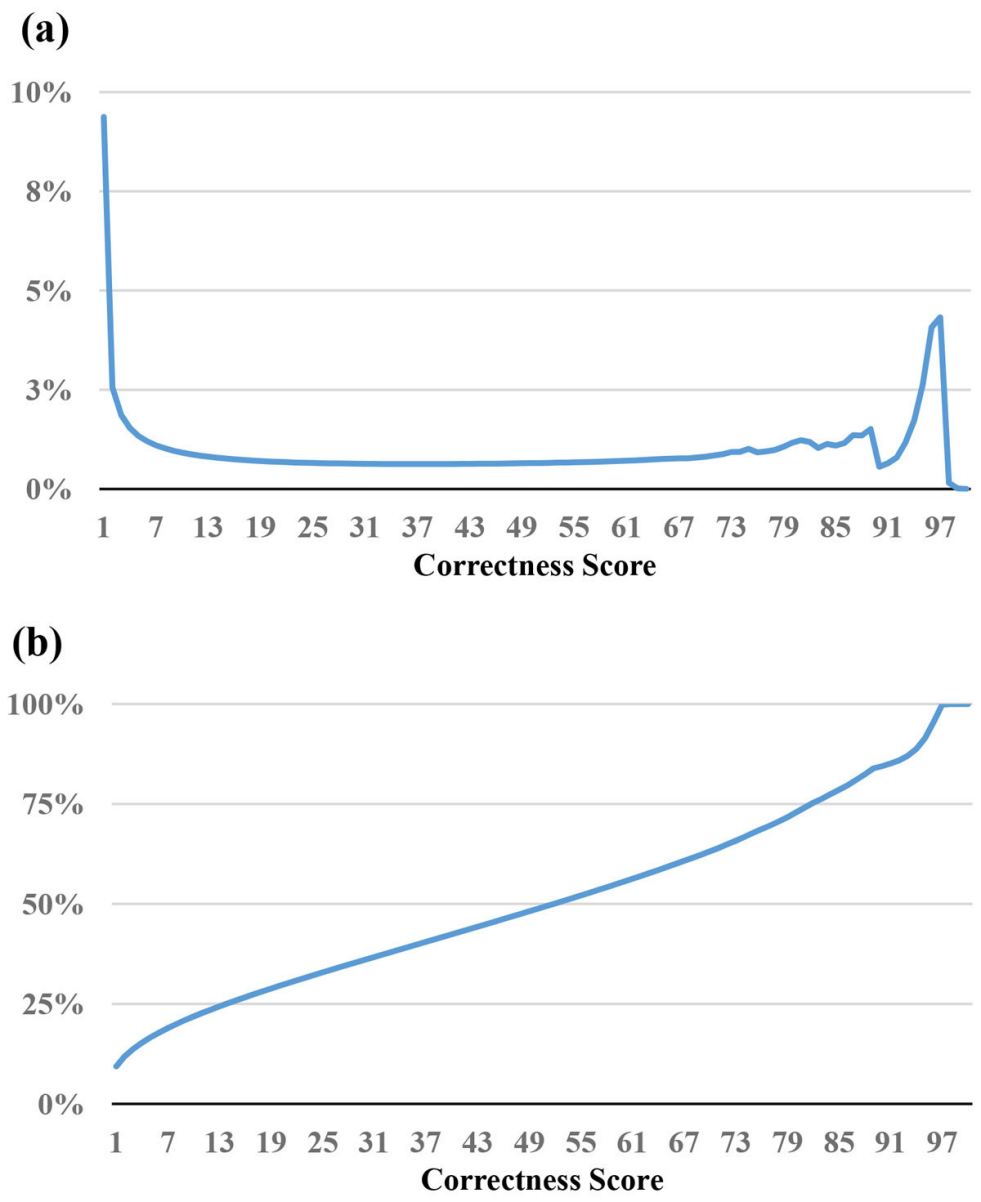
**Statistics of correctness score for the reads in the *B. cereus *dataset**. (a) The percentage of reads for a correctness score. (b) The cumulative percentages of (a).

The read selection strategy, *MinimalQ*, mentioned above only takes the minimal quality value into account. However, bases other than the base with the lowest quality value may also affect the quality of assembly. In this strategy, we take all the bases in the read into consideration. For a base with quality score *Q *of Sanger format (Phred+33), its base-calling error probability *p *is *p *= 10^(-*Q*/10)^. Thus, its "probability of correct identification" is *P_c _*= 1-*p*. *De novo *assemblers are generally divided into two categories: overlap/string graph based assemblers and de Bruijn graph based assemblers [1]. Thus, for a given read of length L in overlap/string graph based assemblers, the probability of a read being correct is the product of the correctness probability *P_c _*(*i*) of every base *i*. We denote the product of *P_c _*(*i*) as ProductQ and calculate ProductQ * 100 as the ProductQ score, For *de Bruijn *graph based assemblers, there are L-k+1 k-mers for a read of length L. The read's correctness score is defined as the minimal value of its k-mer ProductQ scores. Figures 3a and 4a represent the distribution of correctness score of the reads of *E. coli *and *B. cereus*, respectively. Figures 3b and 4b show the percentage of reads filtered out; for example, if we set 10 as threshold, 30% of the *E. coli *reads and 23% of the *B. cereus *reads were filtered out.

#### Results of read subset selection for single-end reads

Our investigation was aimed at determining whether it is possible to select correct reads from raw data, in order to assemble contigs without affecting contig N50 result, achieving high sequence depth with high quality data. In order to determine this, we designed our experiments for single-end reads in three steps.

*Step 1: Different subsets of reads were selected using MinimalQ and ProductQ strategies*.

Step 2: Velvet assembler was used to obtain contigs

Step 3: The assembly results were evaluated using GAGE benchmark

The detailed results are shown in Additional files 5 and 6. The first column of these Tables represents the subsets of reads that were assembled. "Q>*x*" represents reads with minimal quality under *x*, that were filtered out. "PQ>*yy*" represents reads with minimal correctness probability under 0.*yy*, that were filtered out. The reference genomes obtained from NCBI library enabled the calculation of the sequencing depth of the selected reads, and the determination of the percentage of reads left over compared to the original sequence data. The information in the rest of the columns was obtained using GAGE [10] tools. In Additional files 5 and 6, the variances in columns coverage%, #misjoin, and #indel are relatively slight, but the contig N50 and #contig columns, no matter uncorrected or corrected by GAGE, changes significantly.

**Figure 5:**
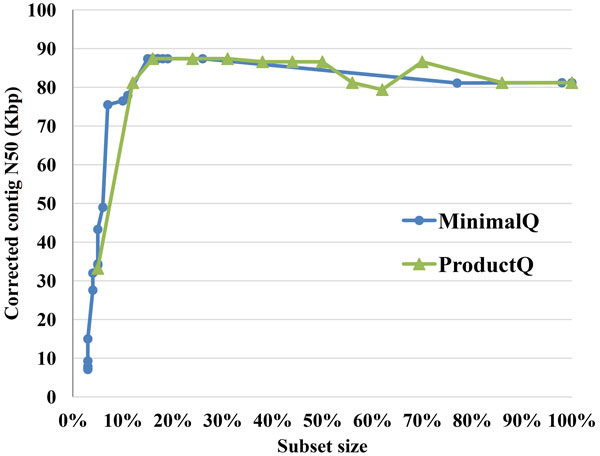
**Corrected contig N50 size vs. subset size of the *E. coli *assemblies using *MinimalQ and ProductQ *strategies**.

**Figure 6:**
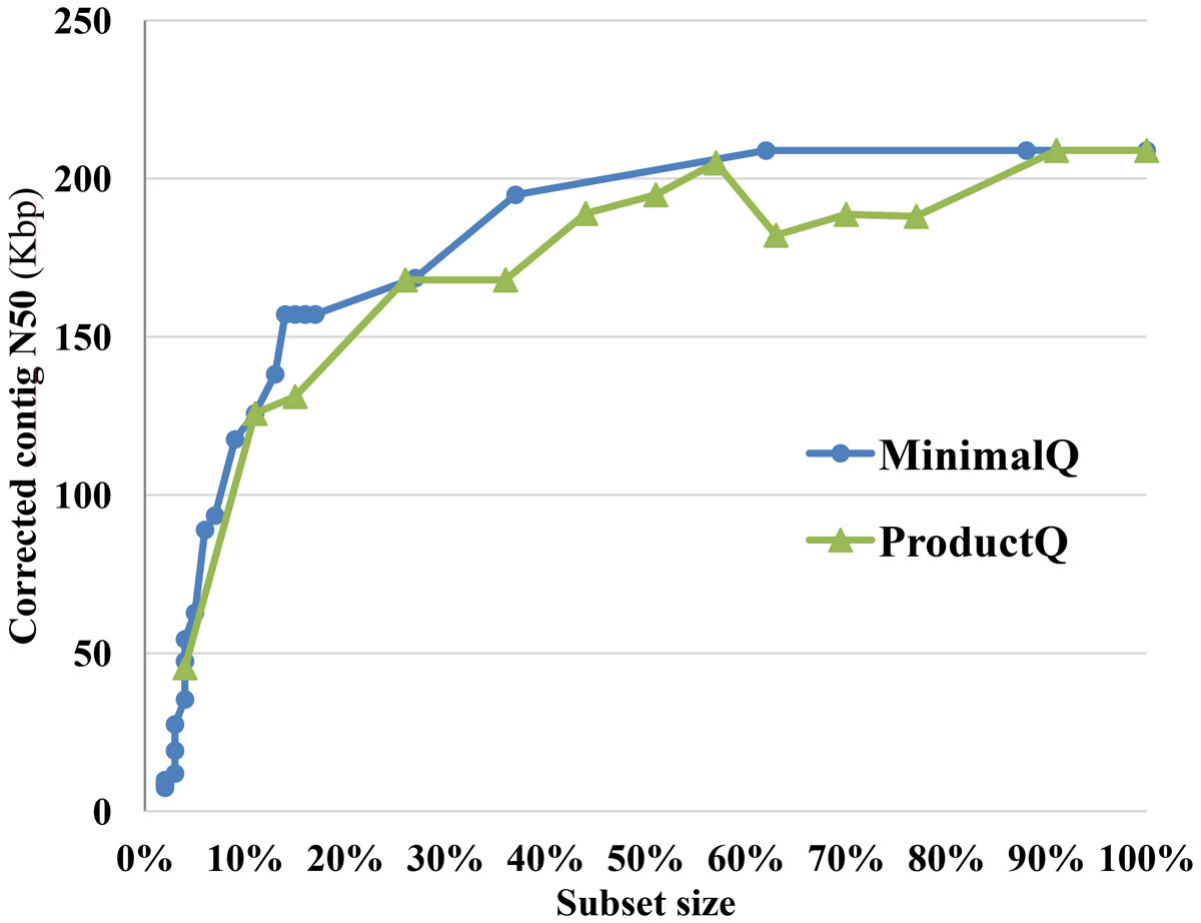
**Corrected contig N50 size vs. subset size of the *B. cereus *assemblies using *MinimalQ and ProductQ *strategies**.

Figure 5 and 6 summarized the results of Tables S2-S3 to analyze the subset sizes with corrected contig N50 for MinimalQ and ProductQ strategies. As shown in Figures 5,6, MinimalQ strategy obtained better corrected contig N50 results comparing to ProductQ strategy in general. We also ran simple random selection of reads and put the results in Additional files 7 and 8 as references. For the *E. coli *dataset, as shown in Figure 5, both MinimalQ and ProductQ strategies can use 20% to 40% of the dataset to obtain the optimal corrected N50 result, which is longer than the corrected N50 of using all the original data and outperformed the corresponding random selection results (Additional file 7). For the *B. cereus *dataset, as shown in Figure 6, the best corrected N50 results occurred at the subset size of 70%-100% for MinimalQ strategy and the subset size of ~90%-100% for ProductQ strategy; besides, in the subset size of 20%-60% for both strategies provided choices to speed up the assemblies with graceful decay of the corrected contig N50. Note that the random selection results for the *B. cereus *dataset (Additional file 8) beat the values at the subset size of 20%-40% in Figure 6 and had similar ranges at the subset size of 40%-60% in Figure 6.

### Subset selection for paired-end reads

PE subset selection selects not only reads but also the paired relations, and it directly affects both results of contigs and scaffolds. A feasible and reasonable way to PE subset selection is to treat a pair of reads as a whole and use the MinimalQ or ProductQ to select the pair is removed or not. Since the constraint of MinimalQ is stricter to obtain an accurate PE subset, we chose MinimalQ as the method for the experiments of PE subset selection. That is, a pair of reads will be selected if the minimal quality value of the two reads is larger than a given threshold. We used the grouper dataset (Table 1) in the experiment of PE selection. We first selected a PE subset of the grouper dataset by MinimalQ, and then used ALLPATHS-LG [11] to assemble the subset into contigs and scaffolds. Since ALLPATHS-LG requires both PE libraries and mate-pair libraries as the input, the five mate-pair libraries mentioned in the Datasets section were also used. To compare genome assemblies of the original set and the subset without the reference genome, we used QUAST [12] for quality assessment.

#### Results of PE subset selection

**Figure 7:**
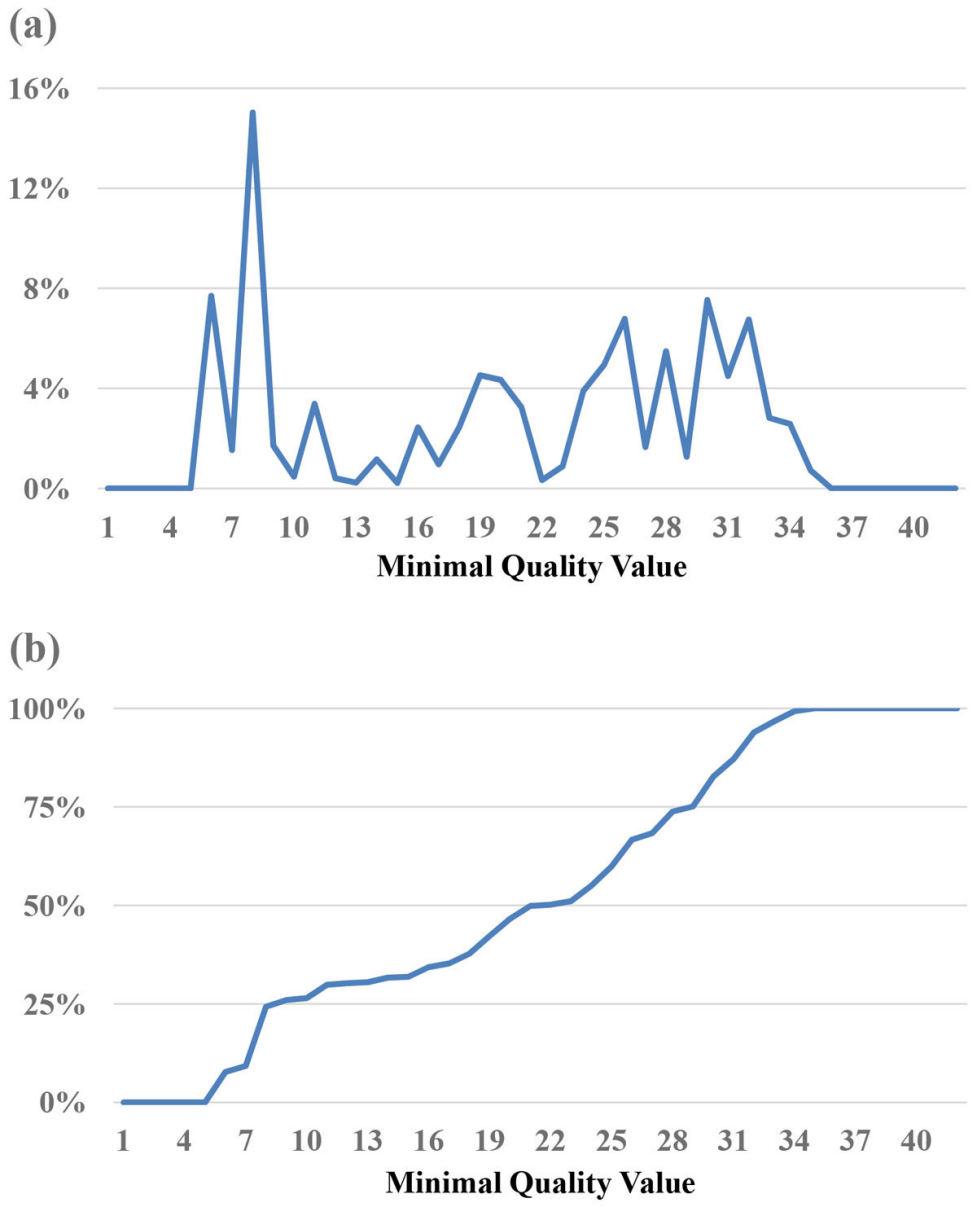
**Statistics of minimal quality value for the PEs in the grouper dataset**. (a) The percentage of PEs for a minimal quality value. (b) The cumulative percentages of (a).

The sequencing depth of the grouper dataset is ~110-120x, which is much smaller than the depths of the *E. coli *and *B. cereus *datasets in Table 1. Referring to [13] and considering the grouper genome is large, we used 60x as the target coverage depth, and consequently set the threshold of MinimalQ as 21 to select the PE subset, which is ~50% of the original dataset, as shown in Figure 7 and Table 2. The mean length of reads of the selected subset is 198.6 bp and is slightly longer than the value of the original set. Comparing the statistics of conigs in Table 2, the subset produced more number of contigs with substantially less contig N50 and less total length of the contigs than those values produced by the original dataset. It seems that the subset selection has no help; however, the results of scaffolds are dramatically different. The largest scaffold of the subset is 21.8 Mbp compared to the original 12.7 Mbp. The N50 scaffold size increased from the original 3354 Kbp to 5443 Kbp of the subset and the number of scaffolds used in the N50 decreased from the original 97 to 61 of the subset. The same trend can also be found for the N75 scaffold size. We also listed the minimal number of the scaffolds whose total length are at or above 1G bp as the last row of Table 2, That is, above 90% of the grouper genome coverage is covered by the top 304 long scaffolds produced by the selected subset, which costs 178 less compared to the original scaffolds. In addition, the original scaffolds contain above 10% more undetermined base 'N'. To compare the scaffolding results for the original dataset and the subset in more details, three figures provided in the QUAST report, i.e., the cumulative length of scaffolds, the N*x *plot of the largest scaffold sizes, and the GC% of scaffolds, are listed as Additional files 9, 10, and 11 respectively. As shown in Additional file 9, the cumulative lengths of the scaffolds produced by the subset obviously are longer than the values of the original scaffolds for the cumulative lengths no larger than 1.05 Gbp. Additional file 10 shows the N*x *scaffold sizes, e.g., N50 and N75 scaffold sizes in Table 2, produced by the subset are substantially larger the corresponding N*x *scaffold sizes of the original scaffolds for most cases. Note that N*x *(where 0≤*x*≤100) is the largest scaffold length, *L*, such that using scaffolds of length ≥ *L *accounts for at least *x*% of the bases of the assembly. Additional file 11 shows the GC content of scaffolds for the two grouper assemblies of the original dataset and the subset are highly similar. As for the runtimes, the assembly of the whole grouper dataset used ~50 days with ~600 GB peak RAM usage on a virtual machine of 32 cores and 1 TB RAM, and the assembly of the PE subset used 7.2 days with ~390 GB peak RAM usage on a physical machine of 40 cores and 1.5TB RAM. Because the two runs of ALLPATHS-LG were at different machines, we cannot compute the ratio but the runtime and RAM usage were greatly reduced. Note that the runtime of getting Figure 7 and generating the selected subset was a few hours.

**Table 2:** Comparing the assembly results of PE subset selection for the grouper dataset.

	Original dataset	Selected subset
*Dataset characteristics*		

Dataset size (G bp)	125	63

# read pairs	319,878,932	158,651,599

Mean length of reads	195.3	198.6

%GC content of reads	41.0%	39.7%

*Assembly statistics *^1^		

# contigs	39,911	53,488

Total contig length	996,203,993	991,109,739

N50 contig size (K bp)	82.2	43.5

# scaffolds	3,917	4,043

Total scaffold length	1,076,396,971	1,062,462,514

Largest scaffold length	12,701,604	21,777,629

N50 scaffold size (K bp) ( L50 number)^2^	3,354 (97 scaffolds)	5,443 (61 scaffolds)

N75 scaffold size (K bp) (L75 number)^2^	1,429 (218 scaffolds)	2,493 (131 scaffolds)

%GC of scaffolds	41.23%	41.17%

# 'N's	79,902,759	71,510,549

# 'N's per 100K bp	7,423.10	6,730.57

# scaffolds for 1G bp^3^	482	304

^1 ^All statistics are based upon the size of contigs and scaffolds both ≥ 1K bp.

^2 ^L50/L75 denotes the minimal number of the scaffolds that produce the 50%/75% bases of the assembly (i.e., all the scaffolds).

^3 ^The minimal number of the scaffolds whose total length ≥ 1G bp.

## Discussion and conclusions

We proposed the subset selection problem of high-depth reads for *de novo *genome assembly and developed two selection strategies, MinimalQ and ProductQ, to select subsets of reads and paired ends. The experiments of read subset selection on two bacteria datasets (Figures 5, 6 and Tables S2-S3) show that both the selection strategies can largely reduce the subset size with graceful decay of the corrected contig N50 and possibly with even better corrected contig N50 sizes. Meanwhile, the results of the experiments of PE subset selection on the grouper data (Table 2) are more promising. It shows that the PE subset reduced much of the runtime and generated substantially longer scaffolds with >10% less unknown bases compared to the original data.

One important issue is how to determine the thresholds of MinimalQ and ProductQ. This issue is affected by multiple factors, including sufficient coverage depths for genome assemblies, characteristics of genome assemblers (e.g., tolerance of variance in coverage depths), and characteristics of datasets and genomes (e.g., read biases and genomic structures that affect assemblies will make the subset selection harder). One feasible solution is to select a small subset initially and perform the assembly to get the contig/scaffold N50. Note that the minimal-quality thresholds of the subset selection methods can be obtained at the *x*-axis of the cumulative-percentage Figures 1b, 2b, 3b, 4b and 7b by choosing a percentage of subset size at the *y*-axis. Then we can relax the thresholds to select until a satisfying contig/scaffold N50 is obtained. To speed up the aforementioned solution, we suggest determining the initial subset sizes by sufficient coverage depths. Desai et al suggest that 50x data is enough to get good genome coverage for assemblies of small and moderate sized genomes [13]. But if the goal is to get the longer contig N50, their results show that the higher depths are still useful. Note that in *de novo *genome assemblies, the genome size is unknown but can be estimated by computing the total number of k-mers in reads divided by the k-mer coverage depth, and then the estimated coverage depth is the total bases of reads divided by the estimated genome size [11].

Despite the aforementioned results already show the usefulness and potentials of the subset selection problem, there are not-yet-solved questions and limitations observed. First, it is difficult to determine the optimal thresholds to get the best subsets producing the best scaffolds/contigs without a certain amount of trial-and-errors. Besides, the functionality of the read selection strategies may be dependent on the datasets involved. For example, we can obtain better corrected N50 using 15% of the original data for the *E. coli *dataset; but for the *B. cereus *dataset, we can only obtain a satisfactory corrected N50 using around 50% of the data. In future work, we will investigate the reasons for the results of the PE subset selection experiments to try to understand how the dataset characteristics and ALLPATHS-LG characteristics affect the results and then improve the subset selection methods. In addition, we plan to integrate the proposed subset selection methods into the CloudDOE software [14] to improve usability.

## Methods

In order to handle large-scale data faster, we developed several tools in Java for preprocessing and analyzing data using MapReduce framework. We developed two pipelines of subset selection for single-end reads, i.e., the MinimalQ pipeline and the ProductQ pipeline. The MinimalQ pipeline contains five main steps, including 1) preprocessing (mentioned in Datasets and Preprocessing), 2) computing MinimalQ values (the *MinimalQ *program), 3) computing the MinimalQ statistics (the *Qstatistics *program), 4) analyzing the statistics and determining the thresholds (mentioned in Results and Discussion), and 5) obtaining the selected subset (the *MinimalQFilter *program). The ProductQ pipeline shares the aforementioned steps 1 and 4, and replaces the steps 2, 3, and 5 with the programs *MinimalProductQ*, *MinimalProductQsta*, and *PQFilter *respectively. The result generated by *MinimalQ *or *MinimalProductQ *program is a list of records, and each record contains a read followed by its corresponding minimal quality value (MQV) or correctness score, respectively. The *Qstatistics *program can produce two types of quality statistics, depending on the input type. It generates the distribution of base quality scores for raw data as the input. For the MinimalQ file as input, the *Qstatistics *program generates the distribution of MQV. The *MinimalProductQsta *program takes *MinimalProductQ *program's output as input and generates the distribution of correctness score. The *MinimalQFilter *program allows users to set a threshold for selecting the reads with MQV above the threshold; similarly, the *PQFilter *program is for selecting the reads with correctness score above a given threshold. Both the outputs of *MinimalQFilter *and *PQFilter *programs are in FASTA format.

For selecting paired-end (PE) reads, we developed two MapReduce programs *PEMQExtractor*, *PEMinimalQFilter *and a single machine program *PEMQsta*. The *PEMQExtractor *program takes shuffled FASTQ data as input to extract a MQV pair for each pair of reads. The *PEMQsta *program reads the MQV pairs, computes the minimal value of each paired MQVs as the PE-MQV, and generates the distribution of PE-MQV. The *PEMinimalQFilter *program allows users to set a threshold for selecting the PE reads with PE-MQVs above the threshold.

## Competing interests

The authors declare that they have no competing interests.

## Authors' contributions

Conceived and designed the experiments: CHF YJC WCC CYL JMH. Performed the experiments: CHF WCC PHH. Analyzed the data: CHF YJC PHH CYL JMH. Wrote the paper: CHF YJC CYL JMH. Developed the software: CHF WCC.

## Supplementary Material

Additional file 1 **Base quality score distribution of the *E. coli *dataset**. (a) Base quality score distribution in ascending order. (b) Cumulative base quality score distribution in descending order. Click here for file

Additional file 2 **Base quality score distribution of the *B. cereus *dataset**. (a) Base quality score distribution in ascending order. (b) Cumulative base quality score distribution in descending order. Click here for file

Additional file 3 **Base quality score distribution of the grouper dataset**. (a) Base quality score distribution in ascending order. (b) Cumulative base quality score distribution in descending order. Click here for file

Additional file 4 **Testing different k values for the Velvet assemblies of the *E. coli *and *B. cereus *datasets**. (a) The *E. coli *dataset. (b) The *B. cereus *dataset. Click here for file

Additional file 5 **Read selection results of the *E. coli *dataset**. (a) Using MinimalQ. (b) Using ProductQ.. Click here for file

Additional file 6 **Read selection results of the *B. cereus *dataset**. (a) Using MinimalQ. (b) Using ProductQ. Click here for file

Additional file 7 **Distribution of corrected contig sizes of the *E. coli *assemblies using the simple random selection**. 102 points were run for the subsets with sizes ranging from 20% to 40% of the original data size. Click here for file

Additional file 8 **Distribution of corrected contig sizes of the *B. cereus *assemblies using the simple random selection**. 117 points were run for the subsets with sizes ranging from 20% to 60% of the original data size. Click here for file

Additional file 9 **Comparison of the cumulative length of scaffolds for the two grouper assemblies of the original dataset and the selected subset**. The *x*-axis denotes the top *x *long scaffolds (ordered from largest (scaffold #1) to smallest). The *y*-axis denotes their cumulative length. The original dataset uses blue curve; the selected subset uses red curve. Click here for file

Additional file 10 **Comparison of the scaffold N*x *for the two grouper assemblies of the original dataset and the selected subset**. N*x *(where 0≤*x*≤100) is the largest scaffold length, *L*, such that using scaffolds of length ≥ *L *accounts for at least *x*% of the bases of the assembly The original dataset uses blue curve; the selected subset uses red curve. Click here for file

Additional file 11 **Comparison of the GC content of scaffolds for the two grouper assemblies of the original dataset and the selected subset**. Scaffolds are broken into nonoverlapping 100 bp windows. The figure shows numbers of windows for each GC percentage. The original dataset uses blue curve; the selected subset uses red curve. Click here for file

## Declarations

Publication charges for this article have been funded by Academia Sinica.

This article has been published as part of *BMC Genomics *Volume 16 Supplement 12, 2015: Joint 26th Genome Informatics Workshop and 14th International Conference on Bioinformatics: Genomics. The full contents of the supplement are available online at http://www.biomedcentral.com/bmcgenomics/supplements/16/S12.
