# Supplementary figures and images for: Subset selection of high-depth next generation sequencing reads for de novo genome assembly using MapReduce framework
Source: BMC Genomics. 2015 Dec 9;16(Suppl 12):S9. doi: 10.1186/1471-2164-16-S12-S9 (PMC4682372; doi:10.1186/1471-2164-16-S12-S9)

**Additional file 1 - Base quality score distribution of the *E. coli* dataset.**

(a)

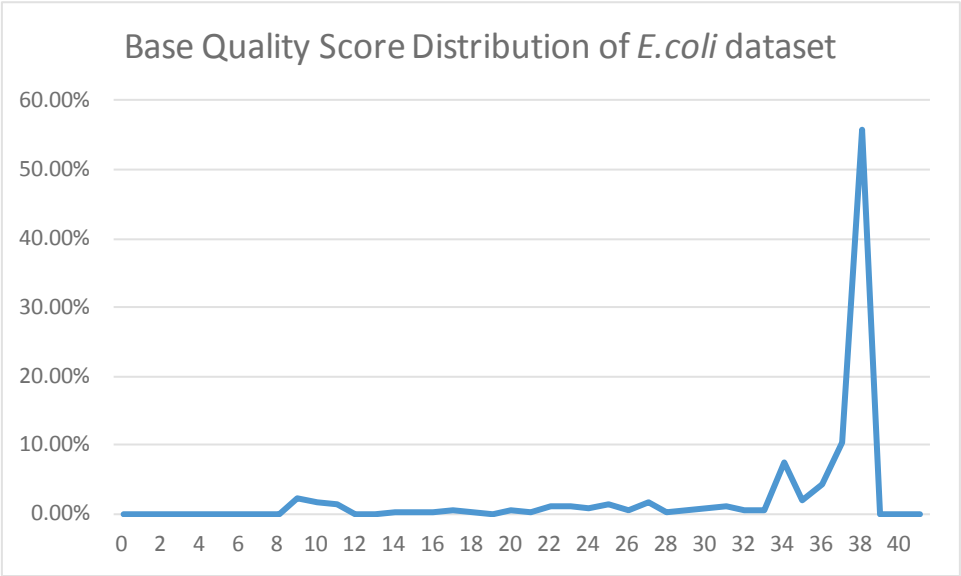

(b)

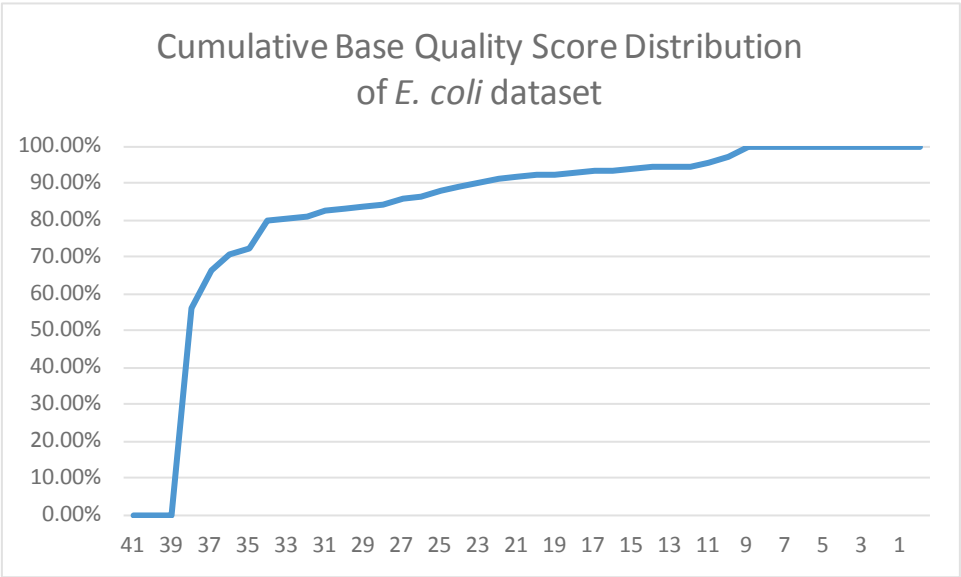

Supplement: Additional file 1 — Base quality score distribution of the E. coli dataset. (a) Base quality score distribution in ascending order. (b) Cumulative base quality score distribution in descending order. [file 1471-2164-16-S12-S9-S1.pdf]

**Additional file 2** - Base quality score distribution of the *B. cereus* dataset.

(a)

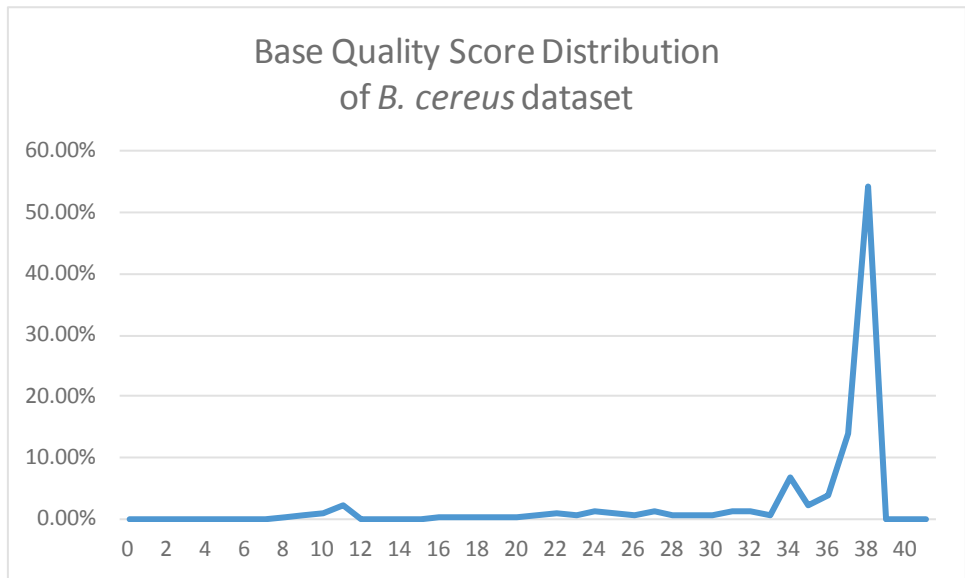

(b)

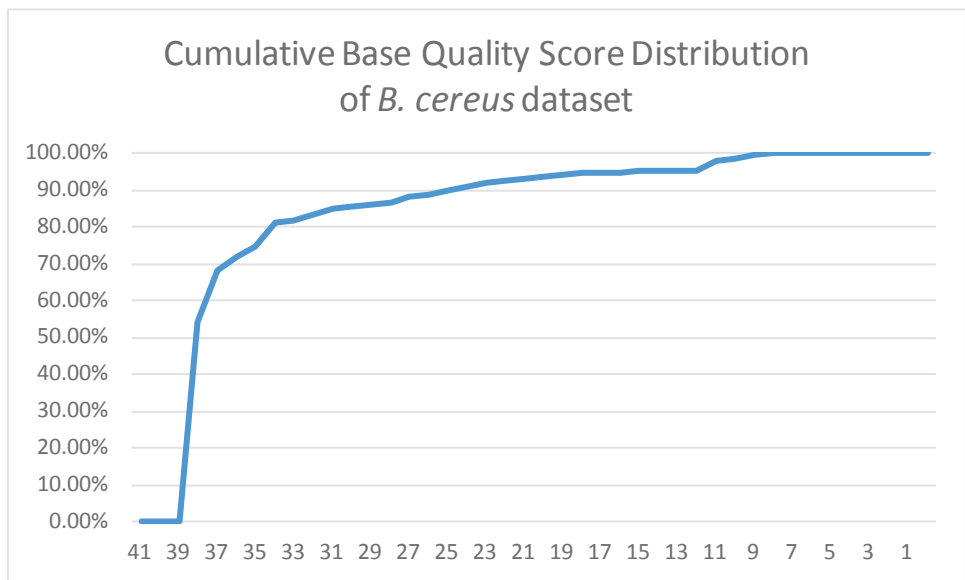

Supplement: Additional file 2 — Base quality score distribution of the B. cereus dataset. (a) Base quality score distribution in ascending order. (b) Cumulative base quality score distribution in descending order. [file 1471-2164-16-S12-S9-S2.pdf]

**Additional file 3 - Base quality score distribution of the grouper dataset.**

(a)

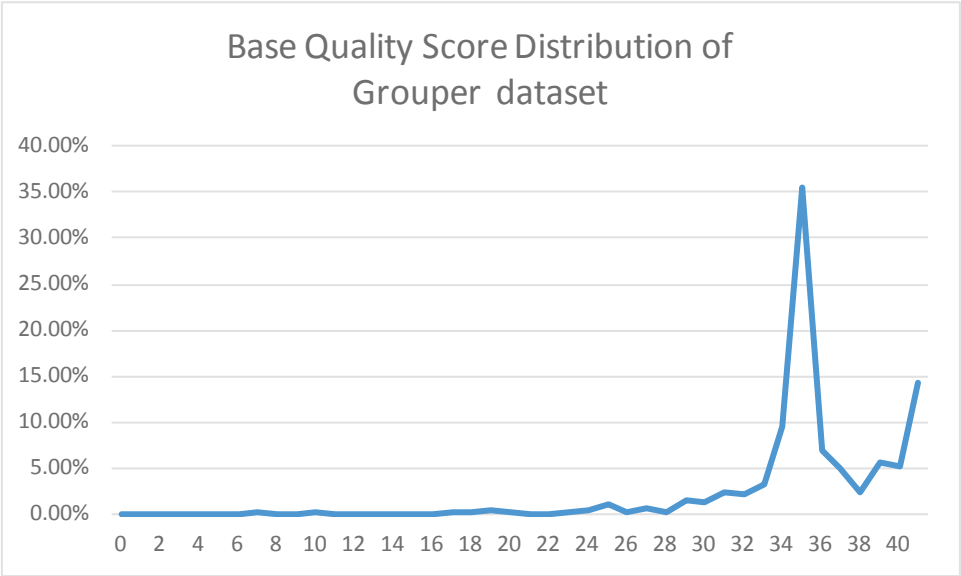

(b)

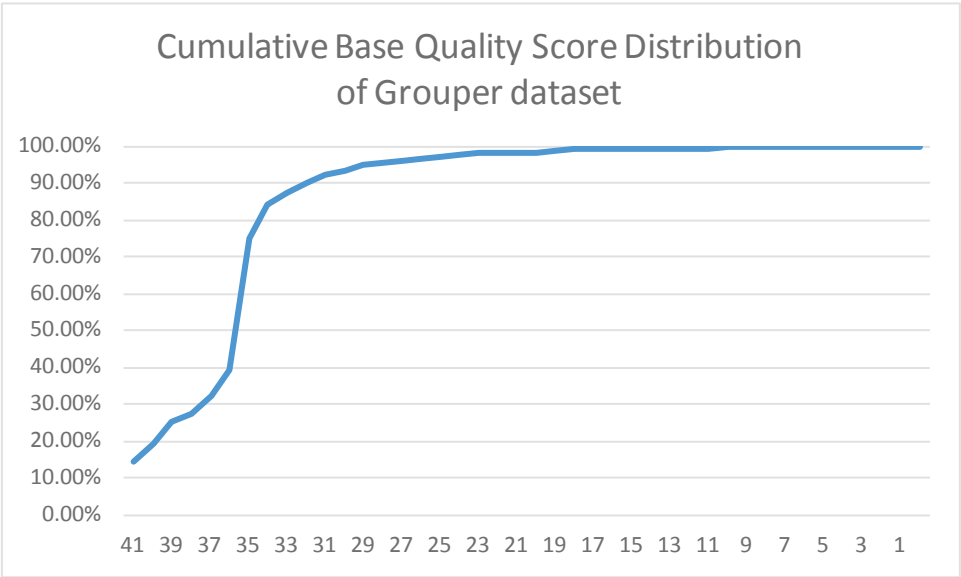

Supplement: Additional file 3 — Base quality score distribution of the grouper dataset. (a) Base quality score distribution in ascending order. (b) Cumulative base quality score distribution in descending order. [file 1471-2164-16-S12-S9-S3.pdf]
