# Supplementary material for: Subset selection of high-depth next generation sequencing reads for de novo genome assembly using MapReduce framework
Source: BMC Genomics. 2015 Dec 9;16(Suppl 12):S9. doi: 10.1186/1471-2164-16-S12-S9 (PMC4682372; doi:10.1186/1471-2164-16-S12-S9)
Supplement: Additional file 4 — Testing different k values for the Velvet assemblies of the E. coli and B. cereus datasets. (a) The E. coli dataset. (b) The B. cereus dataset. [file 1471-2164-16-S12-S9-S4.pdf]

Additional file 4 – Testing different k values for the Velvet assemblies.

(a) The *E. coli* dataset

k	Contig N50	Max contig size
161	161	931
181	181	911
201	10122	58960
221	148577	320117

(b) The *B. cereus* dataset

K	Contig N50	Max contig size
161	161	684
181	181	921
201	201	1598
221	196384	501377
