# Supplementary material for: Subset selection of high-depth next generation sequencing reads for de novo genome assembly using MapReduce framework
Source: BMC Genomics. 2015 Dec 9;16(Suppl 12):S9. doi: 10.1186/1471-2164-16-S12-S9 (PMC4682372; doi:10.1186/1471-2164-16-S12-S9)
Supplement: Additional file 5 — Read selection results of the E. coli dataset. (a) Using MinimalQ. (b) Using ProductQ.. [file 1471-2164-16-S12-S9-S5.pdf]

Additional file 5 - Read selection results of the *E. coli* dataset.

(a) Using MinimalQ

Subset	% Left	Depth (x)	# of contigs	N50 (kb)	# of corrected contigs	Corrected N50 (kb)	#Indels ≥ 5bp	Misjoins	Coverage (%)
Original	100	2853	123	133.9	152	81.2	11	5	99.18
Q>7	98	2785	123	133.9	152	81.2	11	5	99.18
Q>8	77	2185	122	148.8	151	81.1	11	5	99.18
Q>9	26	732	121	133.3	150	87.4	11	6	98.97
Q>10	19	540	120	133.3	150	87.4	11	7	98.97
Q>11	18	499	121	133.3	151	87.4	11	7	98.97
Q>12	17	490	121	133.3	151	87.4	11	7	98.97
Q>13	17	488	121	133.3	151	87.4	11	7	98.97
Q>14	17	476	124	132.8	154	87.4	11	7	98.97
Q>15	16	462	123	132.8	153	87.4	11	7	98.97
Q>16	16	458	124	132.8	153	87.4	11	6	98.97
Q>17	16	449	124	132.8	153	87.4	11	6	98.97
Q>18	15	433	124	132.8	154	87.4	11	7	98.97
Q>19	15	422	124	132.8	154	87.4	11	7	98.97
Q>20	11	307	128	115.0	157	77.9	11	5	98.87
Q>21	10	264	134	105.9	162	76.5	11	6	98.84
Q>22	7	201	138	97.8	169	75.5	11	5	98.85
Q>23	6	163	191	59.2	220	49.0	9	7	98.71
Q>24	5	140	216	53.0	241	43.3	8	4	98.64
Q>25	5	127	242	44.0	265	34.4	8	4	98.17
Q>26	5	123	253	39.2	275	34.1	8	3	98.26
Q>27	4	114	272	36.3	294	32.0	8	3	97.90
Q>28	4	106	313	29.9	334	27.6	8	3	97.43
Q>29	3	94	451	16.6	464	15.0	7	3	96.05
Q>30	3	77	663	9.7	674	9.3	7	3	94.29
Q>31	3	70	768	8.3	775	7.9	7	3	93.75
Q>32	3	68	789	7.9	796	7.3	7	3	93.10
Q>33	3	67	794	7.6	801	7.1	7	3	92.69

(b) Using ProductQ

Subset	%Left	Depth (x)	# of contigs	N50 (kb)	# of cor. contigs	Cor. N50 (kb)	Indels >= 5	misjoins	Coverage (%)
Original	100	2853	123	133.97	152	81.2	11	5	99.18
Score>0	86	2446	126	133.97	157	81.2	11	5	99.18
Score>10	70	2000	119	133.97	152	86.6	11	6	99.18
Score>20	62	1771	120	132.89	153	79.4	11	6	99.18
Score>30	56	1583	125	133.97	157	81.2	11	6	99.18
Score>40	50	1411	124	148.80	156	86.6	11	5	99.18
Score>50	44	1242	121	148.80	152	86.6	11	5	99.18
Score>60	38	1067	122	148.80	153	86.6	11	5	99.16
Score>70	31	875	122	148.80	155	87.4	11	6	99.16
Score>80	24	663	122	133.34	152	87.4	11	6	99.17
Score>90	16	438	123	132.89	152	87.4	11	7	98.97
Score>93	12	327	127	126.13	156	81.2	11	6	98.99
Score>95	5	131	261	43.9	284	33.2	8	4	98.17
