# Supplementary material for: Subset selection of high-depth next generation sequencing reads for de novo genome assembly using MapReduce framework
Source: BMC Genomics. 2015 Dec 9;16(Suppl 12):S9. doi: 10.1186/1471-2164-16-S12-S9 (PMC4682372; doi:10.1186/1471-2164-16-S12-S9)
Supplement: Additional file 6 — Read selection results of the B. cereus dataset. (a) Using MinimalQ. (b) Using ProductQ. [file 1471-2164-16-S12-S9-S6.pdf]

Additional file 6 - Read selection results of the *B. cereus* dataset.

(a) Using MinimalQ

Subset	%Left	Depth (x)	# of contigs	N50 (kb)	# of cor. contigs	cor.N50 (kb)	Indels >= 5	misjoins	Coverage (%)
Original	100	2669	82	208.9	81	208.9	4	2	99.75
Q>7	88	2347	76	208.9	75	208.9	4	2	99.75
Q>8	62	1642	69	208.9	69	208.9	4	1	99.92
Q>9	37	981	70	208.9	71	194.9	4	1	99.89
Q>10	27	717	76	182.3	76	168.6	4	1	99.85
Q>11	17	452	76	168.6	76	157.1	4	1	99.84
Q>12	17	436	77	168.3	78	157.1	4	1	99.84
Q>13	16	433	76	168.6	76	157.1	4	1	99.84
Q>14	16	428	77	168.3	77	157.1	4	1	99.84
Q>15	16	420	77	168.6	77	157.1	4	1	99.84
Q>16	16	412	77	168.6	77	157.1	4	1	99.84
Q>17	15	404	77	168.6	77	157.1	4	1	99.84
Q>18	14	377	78	168.6	78	157.1	4	1	99.84
Q>19	13	332	81	157.1	79	138.2	3	1	99.83
Q>20	11	287	84	138.5	83	125.8	3	1	99.81
Q>21	9	244	89	125.8	87	117.6	3	1	99.77
Q>22	7	174	118	95.6	113	93.5	2	1	99.43
Q>23	6	156	126	89.8	120	89.0	2	1	99.42
Q>24	5	127	159	66.2	155	62.8	2	1	99.00
Q>25	4	114	199	56.4	195	54.4	2	1	98.95
Q>26	4	108	215	48.8	210	47.5	2	1	98.79
Q>27	4	96	267	38.8	262	35.4	2	1	98.25
Q>28	3	88	322	29.3	317	27.5	2	1	97.89
Q>29	3	79	421	20.3	415	19.2	2	1	97.44
Q>30	3	69	612	12.9	601	12.0	2	1	95.94
Q>31	2	62	745	10.8	735	9.9	2	3	95.73
Q>32	2	54	813	8.8	797	8.5	2	1	93.10
Q>33	2	52	988	7.8	968	7.5	2	1	95.05

(b) Using ProductQ

Subset	%Left	Depth (x)	# of contigs	N50 (kb)	# of cor. contigs	Cor.N50(kb)	Indels >= 5	misjoins	Coverage (%)
Original	100	2669	82	208.9	81	208.9	4	2	99.75
Score>0	91	2418	82	208.9	80	208.9	4	1	99.75
Score>10	77	2059	74	188.1	73	188.1	4	1	99.75
Score>20	70	1858	74	188.7	74	188.7	4	1	99.78
Score>30	63	1684	76	182.1	75	182.1	4	1	99.81
Score>40	57	1515	72	205.0	70	205.0	4	1	99.87
Score>50	51	1343	72	208.9	71	194.9	4	1	99.83
Score>60	44	1160	76	191.1	76	189.0	4	1	99.92
Score>70	36	953	75	182.9	75	168.0	4	1	99.90
Score>80	26	684	76	182.9	76	168.0	4	1	99.85
Score>90	15	391	82	138.6	80	131.1	4	1	99.83
Score>93	11	293	87	138.5	84	125.8	3	1	99.81
Score>95	4	222	216	48.6	213	45.4	2	3	98.52
