# Supplementary material for: Subset selection of high-depth next generation sequencing reads for de novo genome assembly using MapReduce framework
Source: BMC Genomics. 2015 Dec 9;16(Suppl 12):S9. doi: 10.1186/1471-2164-16-S12-S9 (PMC4682372; doi:10.1186/1471-2164-16-S12-S9)

Additional file 7 – Distribution of corrected contig sizes of the *E. coli* assemblies using the simple random selection.

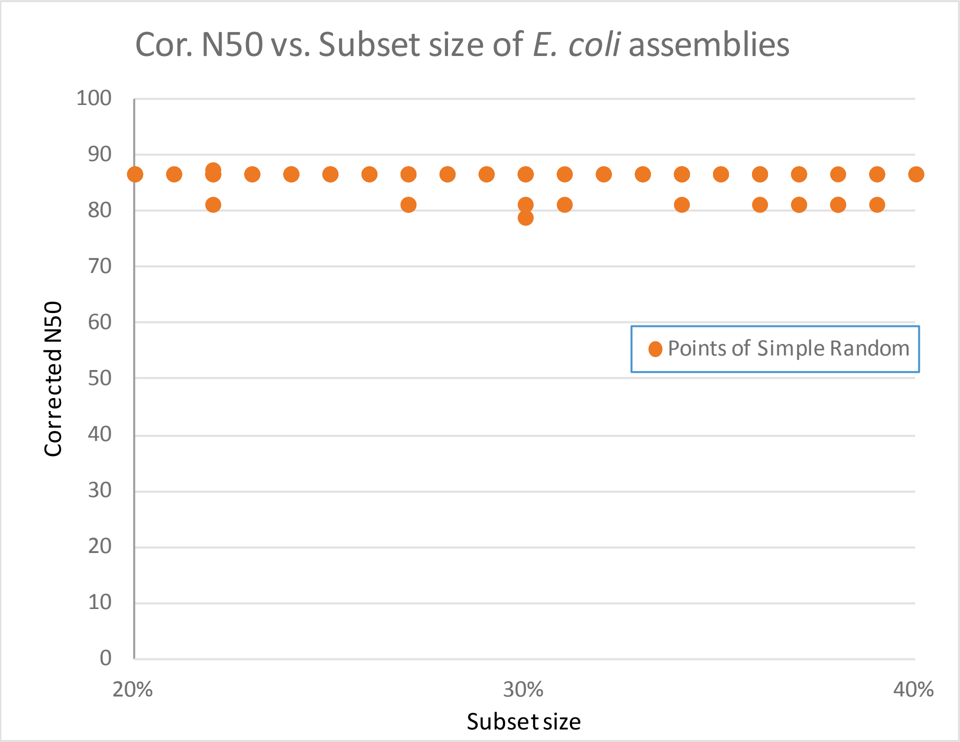

Supplement: Additional file 7 — Distribution of corrected contig sizes of the E. coli assemblies using the simple random selection. 102 points were run for the subsets with sizes ranging from 20% to 40% of the original data size. [file 1471-2164-16-S12-S9-S7.pdf]
