# Supplementary material for: Subset selection of high-depth next generation sequencing reads for de novo genome assembly using MapReduce framework
Source: BMC Genomics. 2015 Dec 9;16(Suppl 12):S9. doi: 10.1186/1471-2164-16-S12-S9 (PMC4682372; doi:10.1186/1471-2164-16-S12-S9)

Additional file 8 – Distribution of corrected contig sizes of the *B. cereus* assemblies using the simple random selection.

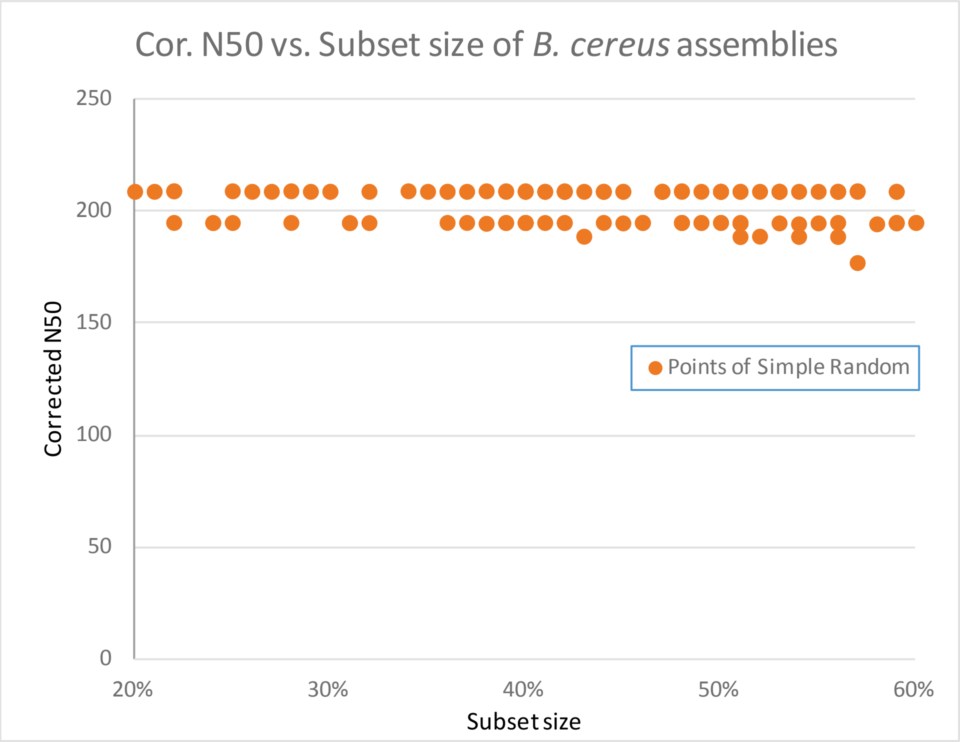

Supplement: Additional file 8 — Distribution of corrected contig sizes of the B. cereus assemblies using the simple random selection. 117 points were run for the subsets with sizes ranging from 20% to 60% of the original data size. [file 1471-2164-16-S12-S9-S8.pdf]
