# Supplementary material for: Subset selection of high-depth next generation sequencing reads for de novo genome assembly using MapReduce framework
Source: BMC Genomics. 2015 Dec 9;16(Suppl 12):S9. doi: 10.1186/1471-2164-16-S12-S9 (PMC4682372; doi:10.1186/1471-2164-16-S12-S9)

Additional file 9 – Comparison of the cumulative length of scaffolds for the two grouper assemblies of the original dataset and the selected subset.

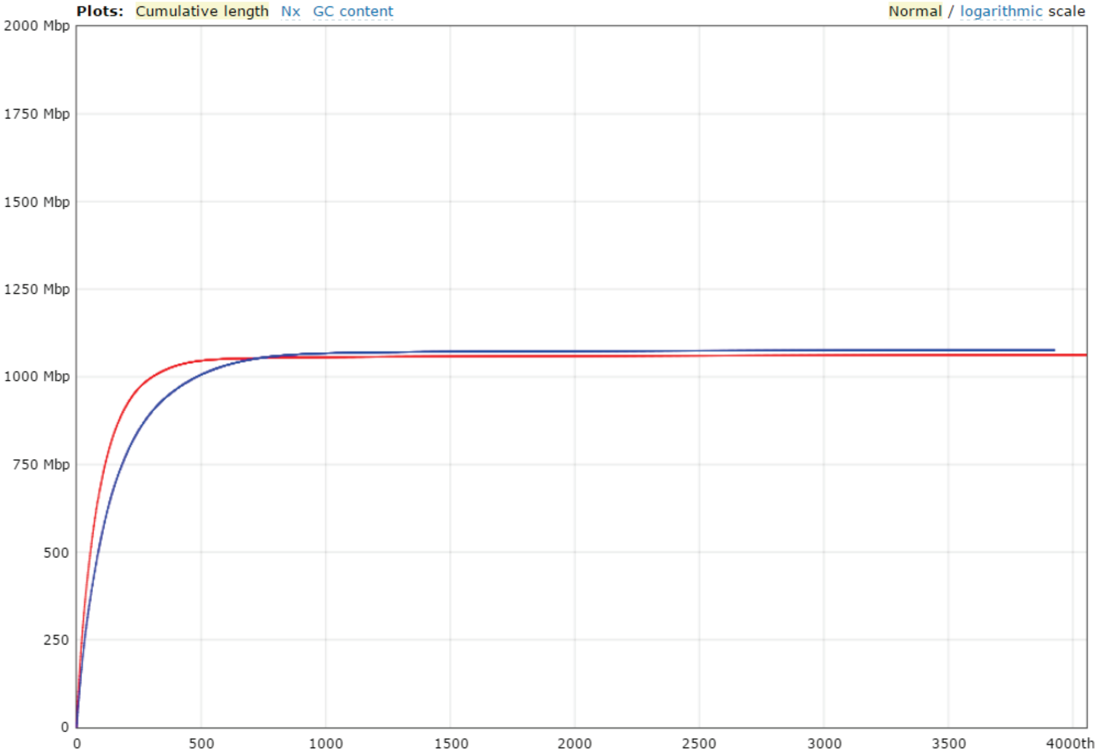

Supplement: Additional file 9 — Comparison of the cumulative length of scaffolds for the two grouper assemblies of the original dataset and the selected subset. The x-axis denotes the top x long scaffolds (ordered from largest (scaffold #1) to smallest). The y-axis denotes their cumulative length. The original dataset uses blue curve; the selected subset uses red curve. [file 1471-2164-16-S12-S9-S9.pdf]
