# Supplementary material for: Subset selection of high-depth next generation sequencing reads for de novo genome assembly using MapReduce framework
Source: BMC Genomics. 2015 Dec 9;16(Suppl 12):S9. doi: 10.1186/1471-2164-16-S12-S9 (PMC4682372; doi:10.1186/1471-2164-16-S12-S9)

Additional file 10 – Comparison of the scaffold Nx for the two grouper assemblies of the original dataset and the selected subset.

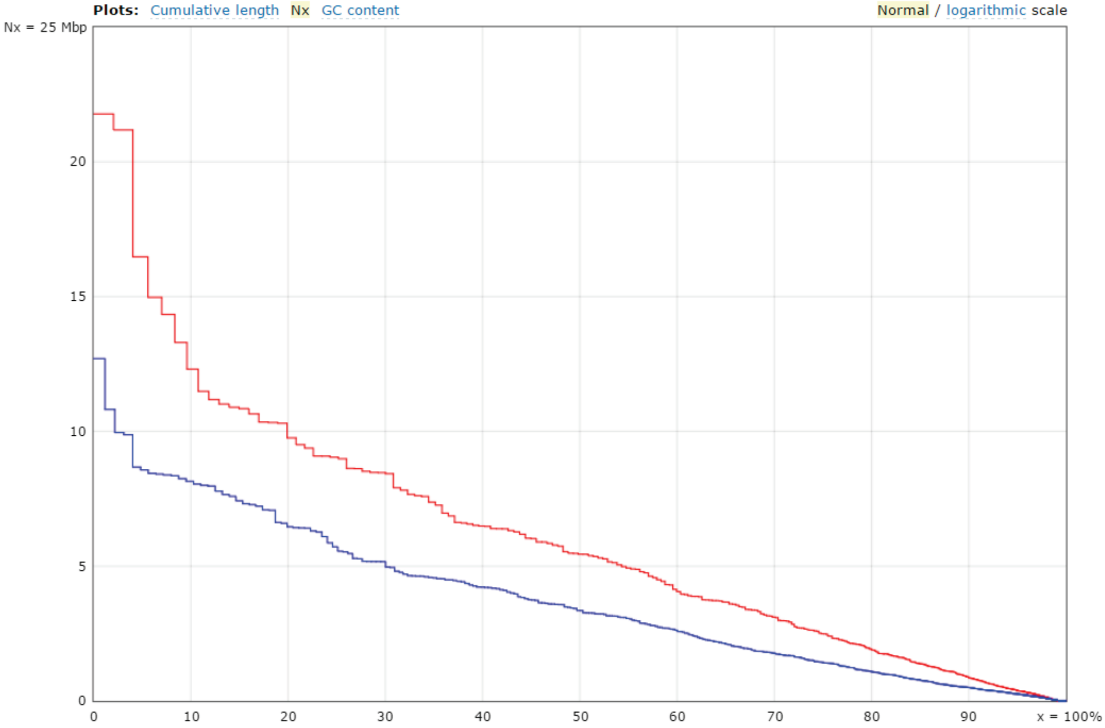

Supplement: Additional file 10 — Comparison of the scaffold Nx for the two grouper assemblies of the original dataset and the selected subset. Nx (where 0≤x≤100) is the largest scaffold length, L, such that using scaffolds of length ≥ L accounts for at least x% of the bases of the assembly The original dataset uses blue curve; the selected subset uses red curve. [file 1471-2164-16-S12-S9-S10.pdf]
