# Supplementary material for: Subset selection of high-depth next generation sequencing reads for de novo genome assembly using MapReduce framework
Source: BMC Genomics. 2015 Dec 9;16(Suppl 12):S9. doi: 10.1186/1471-2164-16-S12-S9 (PMC4682372; doi:10.1186/1471-2164-16-S12-S9)

Additional file 11 – Comparison of the GC content of scaffolds for the two grouper assemblies of the original dataset and the selected subset.

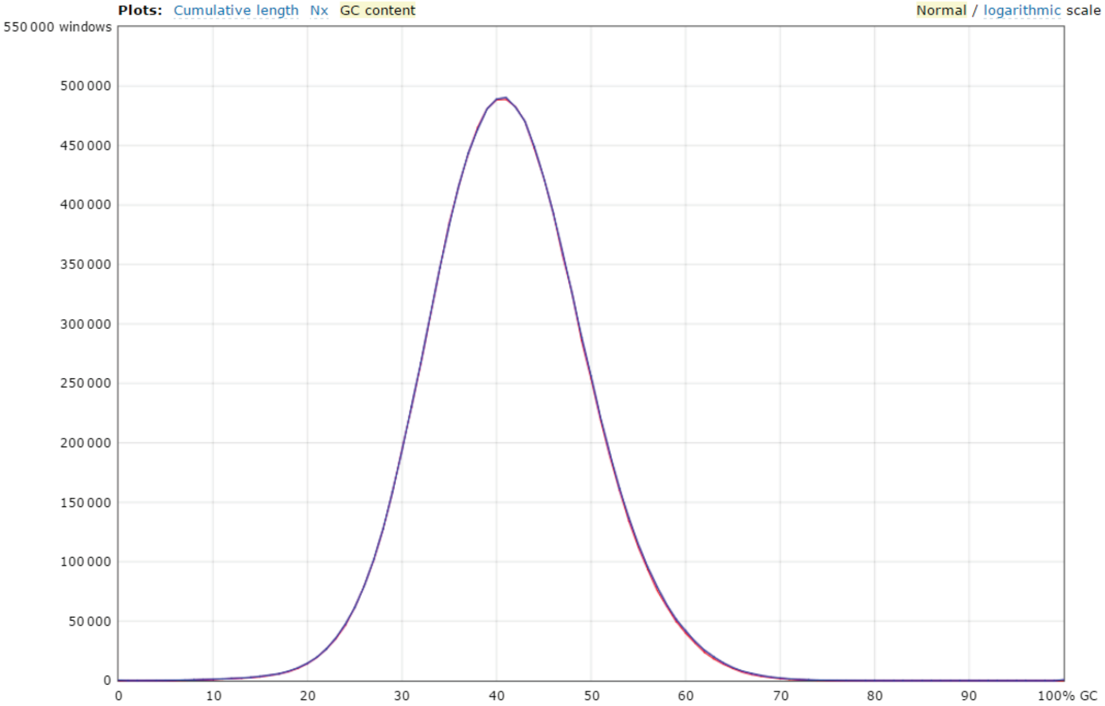

Supplement: Additional file 11 — Comparison of the GC content of scaffolds for the two grouper assemblies of the original dataset and the selected subset. Scaffolds are broken into nonoverlapping 100 bp windows. The figure shows numbers of windows for each GC percentage. The original dataset uses blue curve; the selected subset uses red curve. [file 1471-2164-16-S12-S9-S11.pdf]
